# Toxic Potential of Traditionally Consumed Mushroom Species—A Controversial Continuum with Many Unanswered Questions

**DOI:** 10.3390/toxins12100639

**Published:** 2020-10-02

**Authors:** Petteri Nieminen, Anne-Mari Mustonen

**Affiliations:** 1 Institute of Biomedicine, School of Medicine, Faculty of Health Sciences, University of Eastern Finland, P.O. Box 1627, FI-70211 Kuopio, Finland; anne-mari.mustonen@uef.fi; 2 Department of Environmental and Biological Sciences, Faculty of Science and Forestry, University of Eastern Finland, P.O. Box 111, FI-80101 Joensuu, Finland

**Keywords:** edible mushrooms, mushroom poisoning, mutagenicity, rhabdomyolysis, *Tricholoma equestre*

## Abstract

Mushroom poisonings remain a significant cause of emergency medicine. While there are well-known species, such as *Amanita phalloides*, causing life-threatening poisonings, there is also accumulating evidence of poisonings related to species that have been considered edible and are traditionally consumed. In particular, the *Tricholoma equestre* group was reported to cause myotoxicity. In addition, particular wild mushrooms that are traditionally consumed especially in Asia and Eastern Europe have been subject to suspicion due to possible mutagenicity. Hitherto, the causative agents of these effects often remain to be determined, and toxicity studies have yielded contradictory results. Due to this, there is no consensus about the safety of these species. The issue is further complicated by difficulties in species identification and other possible sources of toxicity, such as microbiological contamination during storage, leading to sometimes opposite conclusions about the edibility of a species. This review focuses on existing data about these types of mushroom poisonings, including the still sparse knowledge about the causative chemical agents. In addition, the aim is to initiate a meta-discussion about the issue and to give some suggestions about how to approach the situation from the viewpoint of the collector, the researcher, and the practicing physician.

## 1. Introduction

Mushroom poisonings cause morbidity worldwide [1,2,3]. Verified or suspected consumption of toxic fungi remains a significant reason to admit patients to emergency departments, and is also an educational challenge for clinicians [1]. This is due to the large number of species with toxic effects, the difficulties in species identification by both patients and hospital personnel, and the ingestion of mushrooms in meals containing several mushroom species simultaneously. In addition, toxicity may be caused by not only the original biological composition of the fruiting body, but by more conventional types of food poisoning, such as incorrect preservation or processing of mushrooms after harvesting [4].

Statistical data about the prevalence of mushroom poisonings are not easily accessible. In Switzerland, approximately 1.4% of consultations about poisonings in general were about mushroom toxicity [5]. While well-known highly toxic mushrooms, such as *Amanita phalloides,* did appear as causative species [2], in many cases the mushrooms suspected to be the agents for morbidity were those that are not considered poisonous, but simply inedible or even edible. In fact, the second most common species reported as the cause in Italy was the sought-after and choice *Boletus edulis* [1]. In a Polish study, a protocol of patient interviewing was combined with identification of causative species by microscopic spore examination [4]. The species appearing as the most common etiological agents were identified as *Armillaria mellea, Macrolepiota procera, Suillus luteus, Cantharellus cibarius,* and *Agaricus campestris*, all recommended as edible by the literature [6]. The number of poisonings caused by edible species reached 400 cases out of a total of 457 [4]. These data indicate that the significance of species that are classified as edible should not be underestimated when examining mushroom poisonings. Obviously, most of these cases may have causes not directly related to the mushroom species or any specific toxic substance. In fact, gastrointestinal problems are common symptoms reported as poisonings when edible mushroom species are concerned. These may be caused, for instance, by high mucus or trehalose content, or incorrect processing of mushrooms after harvesting.

Generally, the question of poisonings related to mushroom species classified as edible has several complex items to discuss. The aim of the present review is to assess some of these issues, as follows:

1. What is the current state of knowledge regarding species considered edible but that have been reported to cause toxic effects?
2. What types of poisonings do these species potentially cause?
3. Are there confounding issues, such as species identification and differences in toxicity of related species?
4. Are there other factors, such as contamination, that complicate the use of species that were previously considered edible?
5. Should scientists, especially the medical and mycological communities, revise the recommendations and warnings considering these species?

Obviously, infectious material can be present in mushrooms, especially if eaten raw or inadequately cooked, for instance in the case of multiple *Listeria monocytogenes* infections with four deaths due to the consumption of enoki mushrooms (*Flammulina velutipes*) in the USA [7]. However, the present review concentrates on the potential toxicity of the mushroom species per se. Thus, more conventional food poisonings due to microbiological contamination (failures in transport and storage) are not of principal focus here. We first present *Tricholoma equestre* as a case study of a species that was previously recommended as edible and delicious, but that has emerged as a potential cause of a novel type of poisoning. Thereafter, other species that are commonly consumed but reported to have adverse effects are introduced. Following this, we shall assess the discussion around these species, especially *T. equestre*, as an example of the complex and problematic aspect of issuing recommendations for mushroom use, with suggestions on how to approach this question from a pragmatic viewpoint.

## 2. From Delicacy to Danger—The Case of *Tricholoma equestre*

Until the 1990s, the yellow tricholoma (*T. equestre* or *T. flavovirens*) was eaten traditionally in large parts of Europe [6,8]. In the 2000s, reports in France [9] and Poland [10] indicated the *T. equestre* group as a cause of severe myotoxicity—that is, delayed rhabdomyolysis (destruction of striated muscle cells) after consumption of large quantities of the species during several consecutive days. Three fatalities were also reported. The symptoms included fatigue, muscle weakness, and myalgia, followed by leg stiffness and dark urine. The laboratory parameters showed clear signs of rhabdomyolysis with elevated plasma creatine kinase (CK) activities, as well as liver function tests with alanine and aspartate aminotransferase activities clearly higher than normal range. In Lithuania, four similar cases and one fatality were reported [11]. The identification of the species was not described in detail in these cases, and thus, we can only assume that it was based on narratives by the patients and comparison with images in mushroom atlases. Gawlikowski et al. [4] identified *T. equestre* as a cause of poisoning in 21 Polish cases. They included spore analysis, but did not list the symptoms of the patients related to this particular species. Although *T. equestre* is also collected in North America, no reports of toxicity have emerged as of 2020 [12].

While the potential causative biochemical agent of the poisonings remains obscure, animal experiments have led to mild but statistically significant elevations in CK activities, indicating rhabdomyolysis, as well as in transaminase activities, reflecting impaired liver function and hepatic toxicity. Bedry et al. [9] were able to trigger elevated CK activities, and tachypnea and muscle fiber disorganization due to *T. equestre* consumption in mice at relatively large doses that would be equivalent to 0.67–1 kg of fresh mushroom per day for three days in human consumption. In further studies on mice fed *T. equestre* at 9 g/kg body mass/day for five days, similar elevations in CK activities were observed [13]. Another in vivo study on mice documented pericardial lymphocyte infiltration and increased CK MB-fraction (CK-MB) that could indicate cardiac muscle damage [14]. Muszyńska et al. [15] studied the biological activities of *T. equestre* identified from sporocarps. They documented potential pro-inflammatory effects in cell culture, and suggested it to be prudent not to harvest and consume this species.

## 3. Are Edible Mushrooms Really Edible?

The issue of mild but statistically significant elevations in enzyme activities (CK, transaminases) in mice caused by *T. equestre* was further complicated when similar effects were induced by other mushroom species with long histories of consumption as delicacies [13,16]. The examined species included *B. edulis*, *Russula* spp., *C. cibarius, Albatrellus ovinus,* and *Leccinum versipelle* dried and fed to laboratory mice. Again, the plasma CK activities increased at 9 g/kg body mass/day, while the histological appearance of striated muscle and liver remained non-diagnostic. It was speculated that the propensity of *T. equestre* to trigger some kind of myotoxicity would not be species-specific, but a phenomenon caused by many edible mushrooms when consumed in large quantities on a daily basis. Regarding cultivated species, there were similar effects on CK activities, and plasma bilirubin concentrations increased without histological findings for *A. bisporus, Lentinula edodes,* and *Pleurotus ostreatus* [17]. The wild (but a cultivated variant of enoki also exists) *F. velutipes* also triggered similar phenomena, including increased CK-MB activities [18]. The possible deleterious effects of long-term consumption are significant to assess for cultivated species, as they are marketed as functional foods to lower elevated blood lipid concentrations [19]. This makes the safety assessment of mushroom consumption complex, as the balance of potentially harmful and beneficial effects has to be taken into account.

A different kind of poisoning by a species frequently consumed especially in Asia is the angel’s wing (*Pleurocybella porrigens*) that caused 17 fatalities due to acute encephalopathy in Japan in 2004 [20]. The poisoning was manifested as demyelination, presumably caused by toxicity targeted at oligodendrocytes. In this case, the toxic effects seemingly required pre-existing conditions, as the victims were mostly hemodialysis patients with chronic renal failure. Other reports of mushroom poisonings triggered by species considered edible are available, but at the moment, the material mostly consists of case studies and remains anecdotal. In many cases, poisonings can result from the misidentification of the species [21]. Furthermore, regional traditions for mushroom preparation can cause discrepancies in the allocation of species into either inedible or good for human consumption. Milk caps (*Lactarius* spp.) are well-known examples of this. In Finnish mushroom atlases, most milk caps are recommended for consumption after boiling [22]. In contrast, *L. rufus*—a species commonly eaten and even commercially harvested in Finland [23]—is considered poisonous in North America [24]. Language barriers and different traditions can thus cause great differences in the official position and layperson attitudes towards a species, and lead to a conclusion that there would be regional differences in toxicity [24].

The issue is further complicated by testing mushrooms for non-acute harmful effects and trace amounts of known toxins. For instance, the highly appreciated species *A. silvaticus, B. edulis,* and *C. cibarius* contain measurable amounts of amatoxins, but at concentrations several orders of magnitude lower than in *A. virosa* [25]. Another type of potentially harmful effect of a widely-eaten mushroom is provided by *L. necator*, a milk cap commonly eaten after boiling [8], that was observed to be highly mutagenic in the *Salmonella* assay [26]. The highly recommended *A. bisporus*, *C. cibarius,* and *B. edulis* also showed mutagenic activity [27]. In addition, *A. bisporus* in the lyophilized form induced tumors in mice, but without any clear dose-response relationship [28], and there are isolated data about suspected rhabdomyolysis caused by the consumption of cultivated *A. bisporus* [29]. Weak mutagenicity was also observed in *P. ostreatus* in canned preparations [30]. This species has been suspected to cause hemorrhagic lesions in several organs, and especially liver inflammation [31]. However, the findings of weak mutagenicity have not led to any general recommendations of withdrawing the above-mentioned species from human consumption. As some potentially harmful effects have been observed in species that are classified as edible, inedible, or poisonous, it is sometimes difficult to establish clear demarcations between edible and poisonous species. Regarding striated muscle damage, *Russula subnigricans,* that is classified as poisonous, triggered rhabdomyolysis in Asia, which was quite similar to the cases reported for *T. equestre* and *A. bisporus* [32,33].

Allergic reactions to mushrooms are also feasible, and for instance, a case study described a young woman developing an anaphylactic reaction after consuming *F. velutipes* [34]. Similarly, *L. edodes* spores have caused hypersensitivity pneumonitis in a mushroom worker [35]. *L. edodes* also displays cholesterol-lowering properties [36] indicating that, again, the balance of beneficial and potentially hazardous effects of a species has to be taken into consideration when assessing its suitability for consumption. Hitherto, the causative agents of these effects often remain to be determined, and scientists have reached contradictory conclusions on edibility, especially regarding *T. equestre* [15,37]. Overall, the existing literature, not only on *T. equestre* but edible mushroom toxicity in general, is relatively scarce and, for obvious reasons, double-blind experiments to study the issue are virtually impossible. This means that, for the most part, evidence regarding edible mushroom toxicity in humans remains anecdotal, which is also reflected in the publication of the reports in relatively low-impact journals.

## 4. Causative Agents of Toxicity in Edible Mushrooms

Unfortunately, the causative chemical agents behind the above-described symptoms can remain elusive. Regarding *T. equestre*, it is likely to have caused rhabdomyolysis [9] and is suspected to be pro-inflammatory [15], but any actual toxic substance and its mechanism of action remain unknown. In some other cases, the substances responsible for the symptoms have been identified, which could provide research hypotheses regarding similar types of effects caused by edible species. For instance, in *R. subnigricans,* which causes rhabdomyolysis similar to *T. equestre*, a highly strained cycloprop-2-ene carboxylic acid was extracted and identified as the toxic molecule [38]. The detailed analytical description of this carboxylic acid allowed us to search for it also in *T. equestre*, but this unstable compound remained elusive. Still, advances are being made. In another related species, *T. terreum*, that is suspected to be toxic, two compounds, saponaceolides B and M have been identified and observed to increase CK levels in mice [39]. Yin et al. [39] did also test *T. equestre* for toxicity in mice and discovered that while the toxic compounds of *T. terreum* extract were present in the non-polar fraction, it was the polar fraction (water layer) of *T. equestre* that proved poisonous. These findings were not too unlike those by Bedry et al. [9], who noticed elevated CK activities associated with the chloroform–methanol lipid-free extract and boiled aqueous extract of *T. equestre*. Unfortunately, Yin et al. [39] were unable to isolate the substance(s) responsible for these effects in *T. equestre*. Still, these findings will give hypotheses for future studies concentrating on the identification of the causative agents in *T. equestre* and other species with suspected myotoxicity.

Toxic compounds have been identified from particular edible and even cultivated species [40]. The nomenclature often follows the scientific names of the species in question. These include ostreolysin from *P. ostreatus*, a 16-kDa protein causing hyperkalemia, bradycardia, and even myocardial ischemia. *P. ostreatus* also contains orellanine, which can generate oxygen radicals and inhibit protein synthesis via its metabolite. Agaritin, from *A. bisporus*, is an L-glutamic acid with DNA-binding potential that also displays mutagenicity. *L. necator* contains necatorin responsible for the observed mutagenic effects in the *Salmonella* assay [41]. Regarding *P. porrigens*, the agent toxic to oligodendrocytes is suspected to be an aziridine-amino acid, a precursor of several cytotoxic amino acids [20]. In *F. velutipes*, flammutoxin, a cardiotoxic protein with hemolytic and edematous effects, is well-documented [42]. Still, all these species remain to be considered edible and even as delicacies when prepared in a proper manner for human consumption. However, in the case of *L. necator*, which has traditionally been consumed mainly in Finland and Eastern Europe, current recommendations include that children and pregnant women should avoid the consumption of this species [22].

Finally, the concentrations of toxins can vary by a great deal, depending on the tissues of the fruiting body, geographical location, and characteristics of the collection site. In the case of *A. phalloides*, the fruiting body caps contain two-fold higher concentrations of *α*-amanitin than the stipes, and several times more than the volvas [43]. It is plausible to assume that similar phenomena would also be present in edible species with diverse biologically active compounds.

## 5. How to Tell Poisonous Species apart from the Safe Ones?

We return to the case of *T. equestre* to consider practical interventions to prevent this type of poisoning in the part of the population that participates in mushroom collecting. Following the report by Bedry et al. [9], an Italian review [44] suggested taking care when mushroom are consumed by the elderly, chronically ill people, pregnant women, and children. A Finnish field guide quite rapidly adopted the position that the consumption of *T. equestre* would be inadvisable [22]. However, the long tradition of consumption without previous reports of toxicity also triggered suggestions of there being no need to change recommendations [45]. The issue has become even more controversial after a new Polish study [37] that reported no harmful effects on humans and suggested that *T. equestre* would be edible if appropriate care would be taken, that is, the mushroom would be consumed in rational amounts by healthy subjects. The authors suggested that the toxic effects could have been caused by individual susceptibility, consumption of morphologically similar species, or by repeated digestion of mushrooms in general.

**Figure 1:**
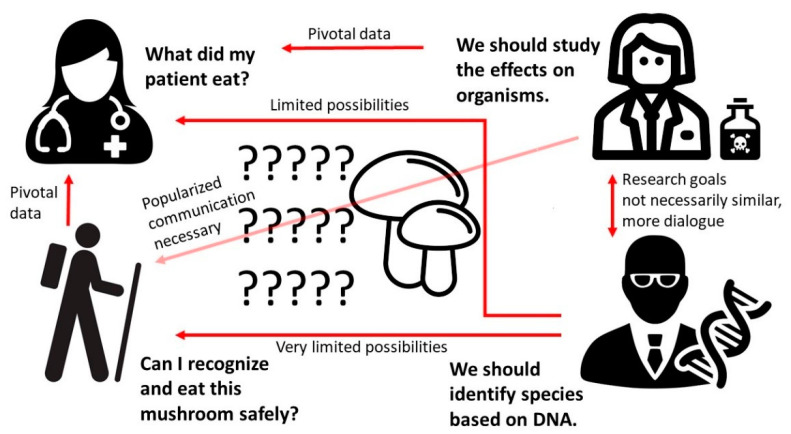
Interest groups of people for whom mushroom consumption has relevance with simplified statements about their positions regarding mushroom toxicity. On the lower left corner, the collector of wild mushrooms needs lay-term information about the safety of the collected fruiting bodies. In addition, she needs information about reliable identification of both edible and potentially harmful species. These data can be provided by mycologists (right panel) but, at the moment, species identification by laypersons cannot rely on spore analyses, nor on genomic studies. The practicing physician on the upper left corner also needs information about the safety and identification methods of the consumed species, but the possibilities of hospitals to conduct either spore or DNA analyses—while plausible—remain limited due to the absence of commercial laboratory kits and/or qualified personnel. Furthermore, the practicing physician needs to communicate with the patient and attempt to gain information about the consumed mushrooms from the collector, whose skills of identification are not based on scientific-level analyses. This leads to uncertainty at the practical level of mushroom consumption (left panel), which should be taken into account when assigning recommendations.

When mushroom poisonings are discussed, there are several viewpoints to consider (Figure 1). First, from the strictly mycological viewpoint, there exist uncertainties about the toxicity. It has been demonstrated quite extensively that a fruiting body that has the macroscopic characteristics of *T. equestre* can cause both human and animal poisonings. However, as discussed by several papers, the identification of the *T. equestre* group of mushrooms requires molecular–phylogenetic analyses [37,46,47,48]. It has also been pointed out that when poisonings were reported, there were no data about the sites where the mushrooms were collected, as soil contaminants, such as heavy metals, can be transmitted to fruiting bodies as toxins. It has been established that depending on the area of collection, edible mushroom species can contain heavy metals at concentrations above the maximum that would be allowed by regulations [49]. In addition, not only the amount of heavy metals but soil characteristics, including texture, pH, and amounts of organic matter and macronutrients, influence the levels of contamination in fruiting bodies [50].

**Figure 2:**
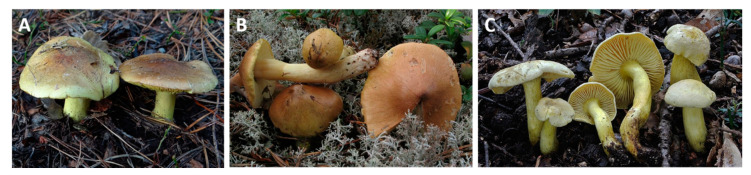
(**A**) *T. equestre* and two visually similar species, (**B**) *T. aestuans*, and (**C**) *T. sulphureum* for comparison. Open source images provided by Creative Commons (https://commons.wikimedia.org/wiki/File:Tricholoma_equestre_G7.jpg by Jerzy Opioła; https://commons.wikimedia.org/wiki/File:Tricholoma_aestuans_25984.jpg by Irene Andersson; https://commons.wikimedia.org/wiki/File:Tricholoma_sulphureum_(Bull.)_P._Kumm 488376.jpg by Nicolò Oppicelli). Visually, the images correspond to species as depicted in mushroom atlases, but the specimens have presumably not been genetically identified, reflecting the real-life situation of the mushroom collectors.

Misidentification is yet another issue. Related inedible but visually quite similar species, such as *T. sulphureum* [6], could be responsible for some of the reported cases regarding *T. equestre* (Figure 2). Another example of complex species determination is the *Pleurotus* (oyster mushroom) group, of which more than one species are commonly cultivated. It is sometimes difficult to determine which type was studied regarding a particular harmful effect, and genetic analyses are necessary to distinguish between related species [51].

The second viewpoint is that of the regular collector of mushrooms who has to decide based on gross morphology if the species is edible or not (Figure 1). This position is represented by Muszyńska et al. [52]. While it is uncertain if the type of *T. equestre* conventionally consumed by collectors is poisonous, the sophisticated and technically complex methods of precise identification (requiring molecular biology) will obviously remain beyond the reach of mushroom collectors. The third viewpoint is held by White et al. [53], who maintain that *T. equestre* consumption is the only possible etiology for the observed cases of sometimes fatal rhabdomyolysis.

Based on these views, it is evident that the precision in species identification that is required for accurate scientific knowledge does not meet the practical needs of laypersons. In this case, we conclude that there is a very high probability that mushrooms that cannot be distinguished from safe forms of *T. equestre* can cause, and have caused toxic syndromes. We also acknowledge that the commonly consumed forms of *T. equestre* would not necessarily be the same as those which were responsible for the observed effects. Furthermore, it is quite well-established that the effects mostly occur after repeated meals, and it could be advisable to restrict the consumption of this species for pregnant women, children, and people with underlying conditions. It is the lack of certainty regarding species identification that means it is impossible to tell at a glance if a fruiting body of *T. equestre* is safe or unsafe to consume. Due to these uncertainties and the different goals of collectors, mushroom systematists, and toxicologists, the discussion around *T. equestre* remains lively. In contrast, while *B. edulis* has induced similar effects on CK levels in mice and humans [13,54], any debate on *B. edulis* or other highly-prized species is absent, and there are no suggestions of them being removed from the list of edible mushrooms. This is presumably due to there being no reported fatalities.

While it is obviously prudent to discuss the potential risks of mushroom consumption, it should be emphasized that the potential benefits of diets that include mushrooms are also many. For example, there is a great amount of data about *F. velutipes* being a good source of biologically active compounds that could have anticancer activities, prevent atherosclerosis, and help to decrease circulating cholesterol levels [55]. This is a reminder that the consumption of the edible mushroom species is much more often safe than hazardous. Still, in our opinion, general recommendations should also include a fourth viewpoint, that of the medical community involved in practical toxicology. From that perspective, the risk of consuming *T. equestre* would not be worth taking, as it is currently impossible to identify the safe individual fruiting bodies by conventional means and it is equally impossible to predict who would experience symptoms and who would become seriously or fatally ill.

## 6. Conclusions

1. The demarcation between poisonous and edible mushroom species is not simple to define, with several edible wild and cultivated species having potential health benefits but also containing toxic compounds.
2. Species considered to be edible can cause allergic reactions (including anaphylaxis), rhabdomyolysis, cardiac toxicity, and mutagenic effects, among others. Mostly, these effects have hitherto been observed in small populations, case studies, laboratory rodents, or in assays on cell cultures.
3. Species identification is a major problem, not only for layperson mushroom collectors but also for scientific studies and in the emergency room. Previous methodology (spore identification) is in some cases inadequate and requires special skills not readily available in hospitals. Thus, genetic testing may be necessary to distinguish between related species that might have very different levels of toxicity.
4. Determining toxicity is further complicated by other causes of food poisoning that may coincide with mushroom ingestion, for instance, microbiological contamination and accumulation of environmental toxins caused by repeated consumption.
5. As the identification of related species is exceedingly complex even in research, it is clear that it is virtually impossible for layperson collectors. Thus, recommendations should be based upon visual recognition, and thus, exclude species that cannot be distinguished from toxic ones by conventional methods. It should be acknowledged that in addition to being an issue of high-quality science and precision cladistics on one hand and of mushroom consumption on the other, edibility also remains a medical issue that has to take into account uncertainty and assess risks based on inadequate data.
